# Impact of Asymmetric Weight Update on Neural Network Training With Tiki-Taka Algorithm

**DOI:** 10.3389/fnins.2021.767953

**Published:** 2022-01-06

**Authors:** Chaeun Lee, Kyungmi Noh, Wonjae Ji, Tayfun Gokmen, Seyoung Kim

**Affiliations:** ^1^Department of Materials Science and Engineering, Pohang University of Science and Technology, Pohang-si, South Korea; ^2^IBM Research AI, Yorktown Heights, NY, United States

**Keywords:** resistive memory, update asymmetry, Tiki-Taka algorithm, neural network, deep learning accelerator, analog AI hardware

## Abstract

Recent progress in novel non-volatile memory-based synaptic device technologies and their feasibility for matrix-vector multiplication (MVM) has ignited active research on implementing analog neural network training accelerators with resistive crosspoint arrays. While significant performance boost as well as area- and power-efficiency is theoretically predicted, the realization of such analog accelerators is largely limited by non-ideal switching characteristics of crosspoint elements. One of the most performance-limiting non-idealities is the conductance update asymmetry which is known to distort the actual weight change values away from the calculation by error back-propagation and, therefore, significantly deteriorates the neural network training performance. To address this issue by an algorithmic remedy, Tiki-Taka algorithm was proposed and shown to be effective for neural network training with asymmetric devices. However, a systematic analysis to reveal the required asymmetry specification to guarantee the neural network performance has been unexplored. Here, we quantitatively analyze the impact of update asymmetry on the neural network training performance when trained with Tiki-Taka algorithm by exploring the space of asymmetry and hyper-parameters and measuring the classification accuracy. We discover that the update asymmetry level of the auxiliary array affects the way the optimizer takes the importance of previous gradients, whereas that of main array affects the frequency of accepting those gradients. We propose a novel calibration method to find the optimal operating point in terms of device and network parameters. By searching over the hyper-parameter space of Tiki-Taka algorithm using interpolation and Gaussian filtering, we find the optimal hyper-parameters efficiently and reveal the optimal range of asymmetry, namely the *asymmetry specification*. Finally, we show that the analysis and calibration method be applicable to spiking neural networks.

## 1. Introduction

Rapid advances in artificial neural network-based machine learning techniques enable significant performance boost in various artificial intelligence (AI) applications exemplified by image classification and natural language processing. As the larger and deeper neural networks trained with more data generally show higher performance in such cognitive tasks, there is an increasing demand for higher computing power and memory bandwidth to support the large number of operations and data processing required for neural network training and inference. Therefore, improving speed and energy-efficiency in AI computing hardware is one of the key challenges to realize more advanced AI applications and to extend the AI application space on low-power systems such as internet of things (IoT) and edge computing devices (Verhelst and Moons, 2017). To address the issue, various optimization techniques such as quantization (Guo, 2018) and compression (Han et al., 2015) are proposed to reduce the size and number of required computations. Along with such techniques for maximizing the efficiency of the existing hardware, there have been efforts to improve the digital hardware architecture (Chen et al., 2020) for energy-efficient AI computing (Zhou et al., 2019).

As an alternative to the existing digital approaches, analog crosspoint array-based neural network computation accelerators have been intensively studied due to the advances in resistive memory device technologies (Haensch et al., 2018; Tsai et al., 2018; Kim et al., 2019b) and their feasibility for various matrix-involved computations such as inversion, eigenvector solving, matrix pseudoinverse (Sun et al., 2019; Wang et al., 2020) and matrix-vector multiplication (MVM). Especially with MVM, by storing weight matrix in the crosspoint array of resistive memory devices and applying voltages corresponding to the input vector values, one can perform fully-parallel neural network computations in analog domain. As the time complexity of MVM with a resistive crosspoint array is approximately *O*(1) (Sun and Huang, 2021), such analog AI hardware is expected to have significant acceleration factor and higher energy efficiency, compared to those of digital counterparts (Agarwal et al., 2016; Gokmen and Vlasov, 2016). While the concept of the resistive crosspoint array-based neural network computation accelerator is promising, the actual implementation of such system has been difficult due to the non-ideal memory device characteristics. One of the major non-idealities which dramatically degrades the system performance is the conductance update asymmetry which indicates the nonidentical amount of up and down conductance changes at a given conductance level (Islam et al., 2019; Xiao et al., 2020). The update asymmetry causes an unexpected dynamic biasing during the training and prevents the weights from reaching the optimum values (Kim et al., 2019a). One obvious direction to resolve this issue is to build a memory device which features symmetric update property. However, the device with an ideal update symmetry is still under development (Lee et al., 2020). The other direction is to develop a special training algorithm which can train the neural network even with asymmetric devices. Tiki-Taka algorithm (Gokmen and Haensch, 2020) resolves the asymmetry issue by adopting an auxiliary array, which stores the information about the history of gradients, along with the main array to store weight values. It is shown that this algorithm minimizes the performance drop caused by update asymmetry and allows robust neural network training even when significant asymmetry presents. However, detailed quantitative study to reveal the relation between degree of update asymmetry and network performance has yet to be explored.

In this work, we dissect the impact of update asymmetry existing in main and auxiliary arrays on neural network performance when Tiki-Taka algorithm is used to train a neural network. We show that update asymmetry in the system is, indeed, a hyper-parameter of the neural network in the optimization point of view, affecting its convergence and performance. Our analysis shows that the auxiliary array is virtually a part of the optimizer to train a neural network; the degree of update asymmetry in the auxiliary array changes the configuration of the optimizer, similar to the case of momentum SGD where a decay factor determines the optimizer (Rumelhart et al., 1986). To further support this point, we compare the performance of different training algorithms on a convex problem and analyze the role of asymmetry in weight optimization process. On the other hand, if device characteristics such as update symmetry work as an hyper-parameter, it is important to fabricate devices with the hardware-side hyper-parameter in the range where the neural network performance is robust against other software-side hyper-parameters. We propose a method to identify the best asymmetry range by introducing a metric, *robustness score*, which provides a concrete rule for comparing the efficiency of training neural networks. By comparing *robustness score* among the different combinations of asymmetry levels in two arrays, it is possible to find the optimal asymmetry range for each array while maximizing the training efficiency and neural network performance. Optimized asymmetry range for each array can relieve the hardware constraints further hence hardware implementation of the algorithm can be accelerated. Finally, our approach can be applied to domains beyond deep neural networks (DNN), including spiking neural networks (SNN) which can be mapped into the resistive crosspoint arrays.

## 2. Preliminaries

### 2.1. Stochastic Gradient Descent With Resistive Crosspoint Array

Stochastic gradient descent (SGD) is a widely-used, first-order optimization method for training neural networks. During forward pass, a neural network infers output and estimates loss by comparing the output with expected values. Based on the loss, gradients of weights in a neural network are calculated during backward pass and later updated during the weight update phase by error back-propagation algorithm. The amount of weight update is proportional to the calculated gradient with the scaling factor called learning rate, η:


(1) $$\begin{array}{l} \begin{array}{cc} w_{ij} & \leftarrow w_{ij}-\eta \nabla_{ij}L=w_{ij}-\eta x_{i}\delta_{j}, \end{array} \end{array}$$

where *w*_*ij*_ indicates a weight from pre-synaptic neuron *i* to post-synaptic neuron *j*, *L* is the loss of a neural network, and ∇_*ij, k*_*L* indicates the derivative of *L* with respect to *w*_*ij*_. *x*_*i*_ is the input activation of pre-synaptic neuron *i*, and δ_*j*_ is the error of post-synaptic neuron *j* propagated from output neurons.

Analog computation with a resistive cross-point array resembles fixed point or quantized number system unlike the digital computation done with floating point number system. For the forward pass, the input activation, *x*_*i*_ is converted to a corresponding discrete voltage, $x_{i}^{q}$, through analog-to-digital converter (ADC). Then, *x*_*i*_ is encoded into a pulse, $PWM\left(x_{i}^{q}\right)$, by pulse width modulation (PWM). As the pulse trains are applied to the rows, currents flowing through the devices are summed at each column line, and one can obtain the weighted sum, *z*_*j*_:


(2) $$\begin{array}{l} z_{j}=\underset{i}{\sum }PWM\left(x_{i}^{q}\right)*g_{ij}, \end{array}$$

where operation “*" includes the integration of currents through the time domain and *g*_*ij*_ is conductance value of *i*^*th*^ column and *j*^*th*^ row device. Then, analog-valued *z*_*j*_ is again quantized into $z_{j}^{q}$ by ADC, and $z_{j}^{q}$ propagates to the next layer as inputs. For the backward pass, the error of pre-synaptic neuron *i*, *e*_*i*_, is calculated in a similar fashion to the errors of post-synaptic neurons, $\delta_{j}^{q}$ :


(3) $$\begin{array}{l} e_{i}=\underset{j}{\sum }PWM\left(\delta_{j}^{q}\right)*g_{ij} \end{array}$$

In the update phase, $x_{i}^{q}$ is converted to a pulse train, $SPG\left(x_{i}^{q}\right)$, by stochastic pulse generator (SPG) for stochastic update of a resistive memory device. In compliance with the rules in Equation (1), the update of *g*_*ij*_ is determined by *x*_*i*_ and δ_*j*_.


(4) $$\begin{array}{l} g_{ij}\leftarrow g_{ij}-\eta x_{i}\delta_{j}^{T}\approx g_{ij}-\sum_{n=1}^{BL}(SPG(\eta_{x}x_{i}^{q})\wedge SPG(\eta_{\delta }\delta_{j}^{q}))\cdot \Delta g_{ij}(g_{ij}), \end{array}$$

where SPG function is defined by pulse bit length (BL) and parameters for pulse probability, satisfying η = η_*x*_η_δ_. η_*x*_ and η_δ_ can be modulated, such that two SPGs will have similar pulse generating probability (Gokmen and Vlasov, 2016; Gokmen et al., 2017). Δ*g*_*ij*_(*g*_*ij*_) is the amount of conductance change of *g*_*ij*_ as a function of *g*_*ij*_, which is dependent on the update characteristics of the specific memory device. Here, we assume a linear model for Δ*g*_*ij*_(*g*_*ij*_) which is described in section 2.2.

When mapping a neural network into resistive cross-point arrays for analog operations, we note that each weight can be expressed with a single or multiple memory cells depending on the architecture or weight mapping scheme (Xiao et al., 2020). As an example, a pair of memory cells, *g*_*ij*_ and *g*_*ij, ref*_, can be read differentially to represent a single weight: *w*_*ij*_ = *K*(*g*_*ij*_−*g*_*ij, ref*_), where *K* is a factor that scales the weight into the conductance (Gokmen and Haensch, 2020).

### 2.2. Asymmetric Conductance Update in Resistive Memory Devices

**Figure 1:**
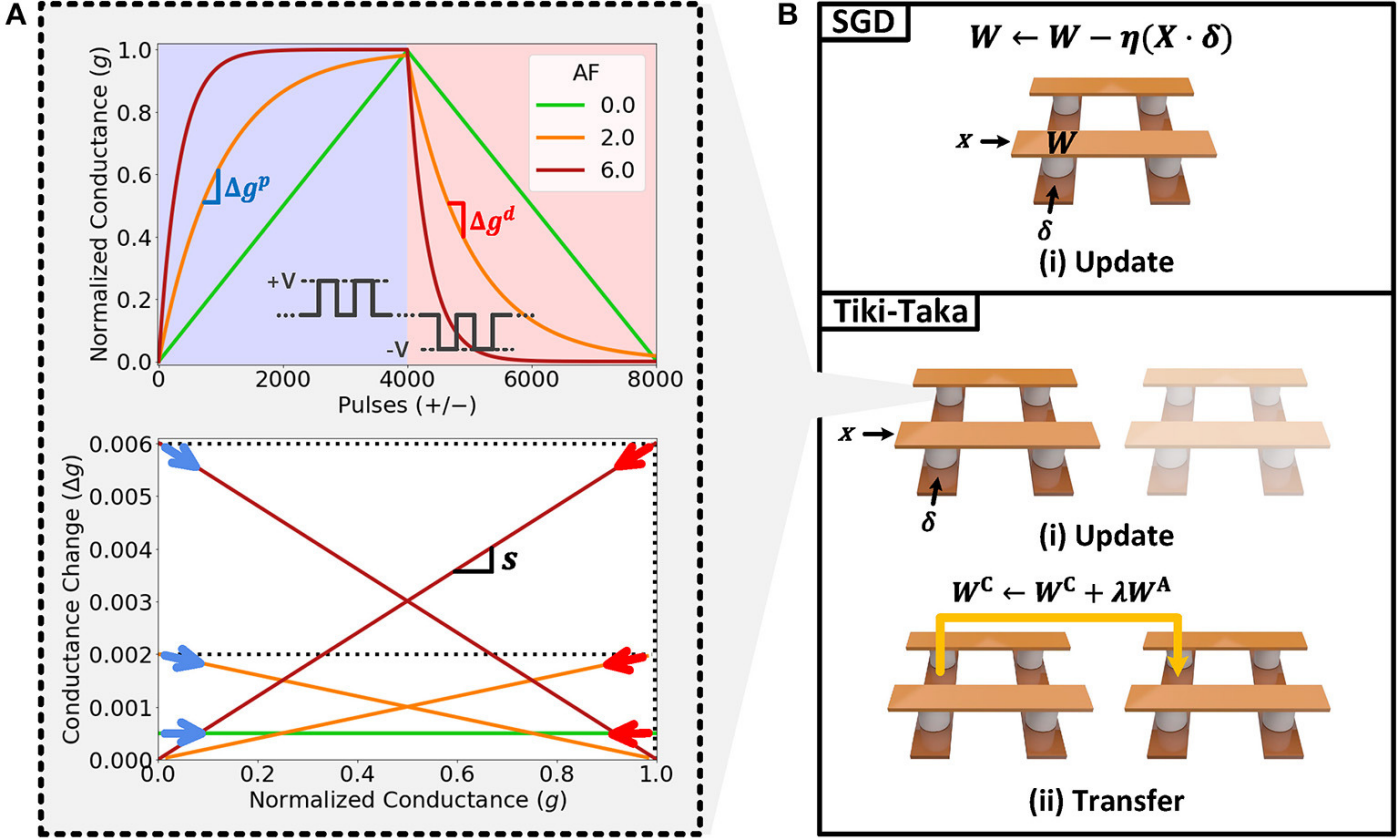
**(A)** Normalized conductance of three resistive memory devices with different AF values as a function of pulse number when 4,000 positive pulses (+*V*) and 4,000 negative pulses (−*V*) are applied. The conductance change (Δ*g*) with respect to the normalized conductance (*g*) is shown below. (see Supplementary Materials section 3.1 for the details of experimental environments.) **(B)** Comparison of conductance update process between SGD and Tiki-Taka algorithm. In case of SGD, an array, *W*, is updated with two parameters, *x* and δ in the update phase. In Tiki-Taka algorithm, there are two steps: (1) update and (2) occasional transfer. In the update phase, like SGD, the auxiliary array, *W*^*A*^, is updated. In the following transfer phase, its state is occasionally transferred and updates *W*^*C*^.

Practical resistive memory devices feature various types of non-ideal characteristics such as asymmetric conductance response (or update asymmetry), short retention time, device-to-device variation and limited number of states. Among them, update asymmetry is known to hamper the convergence (Huang et al., 2020) of SGD-based neural network training and causes a significant performance drop. As shown in Equation (4), it is the Δ*g*_*ij*_(*g*_*ij*_) term which distorts the actual update from the ideal update behavior defined in Equation (1). That is, *g*_*ij*_ will represent a weight value distorted form ideal software calculation after several cycles of weight update unless Δ*g*_*ij*_(*g*_*ij*_) is unity. For most variants of resistive memory devices, as illustrated in Figure 1A, Δ*g*_*ij*_(*g*_*ij*_) scales up or down the amounts of updates, which results in inconsistent updates of the conductance of devices (or weights). This type of behavior not only misguides the direction of updates, but also reduces the effective number of states in resistive memory devices.

Figure 1A illustrates the conductance(*g*) and the conductance change behavior (Δ*g*) which can be described by the following equations, Equation (5):


(5) $$\begin{array}{l} g_{ij}\leftarrow g_{ij}+\Delta g_{ij} \end{array}$$

(6) $$\begin{array}{l} \Delta g_{ij}=d_{ij}\cdot (s_{ij}\cdot g_{ij}+\Delta g_{0,ij})\\ \;=\{\begin{array}{l} d_{ij}\cdot \left(\frac{\Delta g_{0,ij}^{+}}{g_{max,ij}}\cdot g_{ij}+\Delta g_{0,ij}^{+}\right)\text{when }\Delta g_{ij}>\text{0}\\ d_{ij}\cdot \left(\frac{\Delta g_{0,ij}^{-}}{g_{min,ij}}\cdot g_{ij}+\Delta g_{0,ij}^{-}\right)\text{ when }\Delta g_{ij}<\text{0} \end{array}\\ \; \end{array}$$

*g*_*min, ij*_ and *g*_*max, ij*_ is the minimum and maximum conductance state of resistive device and $\Delta g_{0,ij}^{\pm }$ is the amount of Δ*g*_*ij*_ when *g*_*ij*_ of the device is at *g*_0, *ij*_ = (*g*_*max, ij*_+*g*_*min, ij*_)/2. *s*_*ij*_, namely *slope*, is defined as $\Delta g_{0,ij}^{\pm }/g_{max\left(min\right),ij}$ for nonlinear device, which represents the reciprocal of approximate number of states of the device. For ideal device, i.e., perfectly symmetric and linear device, *slope* is defined zero. *d*_*ij,k*_ is number of pulse needed to achieve desired amount of *w*_*ij,k*_ (see Supplementary Materials section 1.1 for the details).

To embody the update asymmetry of a device, we introduce a parameter called *asymmetry factor*, AF(>0), which is associated with existing parameters: $\Delta g_{0,ij}={\text{AF}}_{ij}\cdot \Delta {\hat{g}}_{0,ij}$, where $\Delta {\hat{g}}_{0,ij}$ is a unit amount of Δ*g*_0, *ij*_. Thus, Equation (6) can be rewritten into Equation (7):


(7) $$\begin{array}{l} \Delta g_{ij}=\{\begin{array}{cc} d_{ij}\cdot \left({\text{AF}}_{ij}\cdot \Delta {\hat{g}}_{0,ij}^{+}\right)\cdot \left(\frac{1}{g_{max,ij}}\cdot g_{ij}+1\right) & \text{when }\Delta g_{ij}>0\\ d_{ij}\cdot \left({\text{AF}}_{ij}\cdot \Delta {\hat{g}}_{0,ij}^{-}\right)\cdot \left(\frac{1}{g_{min,ij}}\cdot g_{ij}+1\right) & \text{when }\Delta g_{ij}<0 \end{array} \end{array}$$

Equation (7) shows that symmetry of device is solely defined with $AF\cdot \Delta {\hat{g}}_{0,ij}$, if *g*_*min, ij*_, *g*_*max, ij*_ and $\Delta {\hat{g}}_{0,ij}^{\pm }$ is fixed. Therefore, we can compare the asymmetry of device within the same range by modulating AF. In the case of ideal device, *slope* equals zero and Δ*g*_0, *ij*_ is constant regardless of *AF*_*ij*_. However, to make the notation consistent, we denote this case as *AF*_*ij*_ = 0. For instance, there are three different cases in Figure 1A, namely, *AF*_*ij*_ = 0;*AF*_*ij*_ = 2.0;and*AF*_*ij*_ = 6.0. All three cases have the same *g*_*min, ij*_, *g*_*max, ij*_ and $\Delta {\hat{g}}_{0,ij}$. We note that many devices show non-zero AF behavior (Brivio et al., 2018; van De Burgt et al., 2018; Islam et al., 2019; Kim et al., 2019b).

### 2.3. Tiki-Taka Algorithm

Tiki-Taka algorithm (Gokmen and Haensch, 2020) is proposed to resolve the performance degradation issue in neural network training caused by the update asymmetry in the crosspoint elements. We showed that the asymmetric update prevents devices from being accurately updated by the amount of calculated gradients. Figure 1B illustrates the key difference between SGD and Tiki-Taka algorithm in the weight update phase. In the case of SGD, an array, *W*, stores the weight vectors of a neural network. In the update phase, a weight, *w*_*ij*_, is updated with the gradient ∇_*ij*_*L*(= *x*_*i*_δ_*j*_). On the other hand, Tiki-Taka algorithm requires one additional array, namely *A*, and it stores Δ*W* by accumulating gradient vectors, ∇*L*. The weight vectors stored in the array *A* are denoted as *W*^*A*^ and the array, *C*, stores the weight vectors denoted as *W*^*C*^. When compared to SGD, Tiki-Taka algorithm accumulates the gradient, ∇_*ij*_*L*, in $w_{ij}^{A}$. Then, the accumulated gradient is transferred to update the weight, $w_{ij}^{C}$ at every *ns* steps. Tiki-Taka algorithm supports sparse update by adopting cell-selection vector, **u**_π_, where **u**_π_(*i*) is the *i*^*th*^ element of a sparse vector, **u**, such as a one-hot vector and π is a permutation, π:{1, ⋯ , *n*} → {1, ⋯ , *n*}. Finally, Tiki-Taka algorithm allows the array *A* to participate in the forward pass by introducing a parameter, γ∈[0, 1]: $w_{ij}=\gamma w_{ij}^{A}+w_{ij}^{C}$. Therefore, the effective weight *w*_*ij*_ depends on γ: if γ = 1, $w_{ij}^{A}+w_{ij}^{C}$ replace the effective weight vector. Otherwise, if γ = 0, then the effective weight is $w_{ij}=w_{ij}^{C}$. In summary, Tiki-Taka algorithm can be formulated as follows:


(8) $$\begin{array}{l} w_{ij}^{A}\leftarrow w_{ij}^{A}-\eta \nabla_{ij}^{\gamma }L \end{array}$$

(9) $$\begin{array}{l} w_{ij}^{C}\leftarrow \{\begin{array}{ll} w_{ij}^{C}+\lambda w_{ij}^{A}\cdot u_{\pi }(i) & \text{, every }ns\text{ step}\\ w_{ij}^{C} & \text{, otherwise} \end{array}, \end{array}$$

where $\nabla_{ij}^{\gamma }L$ indicates the derivative of the *L* with respect to $w_{ij}=\gamma w_{ij}^{A}+w_{ij}^{C}$. η and λ indicates SGD learning rate and transfer learning rate, respectively.

## 3. Tiki-Taka Algorithm and Update Asymmetry

### 3.1. Mismatch Factor and Weight Update Formulation

Here, we formulate the impact of asymmetry by adopting even-odd function decomposition, with which any function can be represented (Gokmen and Haensch, 2020), and integrate the model into the SGD update rule in Equation (1) (see Supplementary Materials section 1.2 for the details):


(10) $$\begin{array}{l} \begin{array}{c} w_{ij}\leftarrow w_{ij}-\eta \nabla_{ij}L\cdot Sym\left(w_{ij}\right)+\eta |\nabla_{ij}L|\cdot Asym\left(w_{ij}\right)\\ Sym\left(w_{ij}\right)=AF_{ij},\;Asym\left(w_{ij}\right)=AF_{ij}\frac{w_{ij}}{w_{max,ij}} \end{array} \end{array}$$

where the function *Sym* represents symmetry of the devices and the function *Asym* corresponds to asymmetry. The weight, *w*_*ij*_, is scaled with a factor *K* from the conductance, *g*_*ij*_: *w*_*ij*_ = *K*·(*g*_*ij*_−*g*_*ref, ij*_) and $\Delta {\hat{w}}_{0,ij}=K\cdot \Delta {\hat{g}}_{0,ij}$. *w*_*max, ij*_ is a correspond weight to the maximum amount of conductance, *g*_*max, ij*_. To analyze it in the aspect of optimization, Equation (10) is reformulated as follows:


(11) $$\begin{array}{l} \begin{array}{l} w_{ij}\leftarrow w_{ij}-\eta \nabla_{ij}L\cdot Sym\left(w_{ij}\right)+\eta |\nabla_{ij}L|\cdot Asym\left(w_{ij}\right)\\ \;=w_{ij}-\eta \nabla_{ij}L\cdot \left(Sym\left(w_{ij}\right)+\text{sgn}\left(\nabla_{ij}L\right)\cdot Asym\left(w_{ij}\right)\right)\\ \;=w_{ij}-\eta \nabla_{ij}L\cdot MF\left(w_{ij},\text{sgn}\left(\nabla_{ij}L\right)\right) \end{array} \end{array}$$

** Definition 3.1 (Mismatch factor (MF))**. *Mismatch factor (MF) is an additional factor appeared in the original SGD weight update rule, originated from the update asymmetry of the weight storage device:*


(12) $$\begin{array}{l} MF_{ij}=MF_{ij}(w_{ij},sgn(\nabla_{ij}L))=Sym(w_{ij})+sgn(\nabla_{ij}L)\cdot Asym(w_{ij})\\ \;=AF_{ij}\cdot \left(1+sgn(\nabla_{ij}L)\cdot \frac{w_{ij}}{w_{max,ij}}\right) \end{array}$$

*MF* is multiplied to the gradient of a weight, ∇_*ij*_*L*, and affects the weight optimization process. As observed in the Definition 3.1, *MF*_*ij*_ scales with *AF*, and is dependent on the sign of gradient and the current weight state, *w*_*ij*_, of a device. The dependence on the sign of gradient can create fluctuation of *MF*_*ij*_ if sgn(∇_*ij*_*L*) changes the sign, and *AF*_*ij*_ magnifies the fluctuation of *MF*_*ij*_. Therefore, there can be abrupt change in *MF*_*ij*_ affecting the neural network training.

### 3.2. Impact of Asymmetry: Case Studies

**Figure 2:**
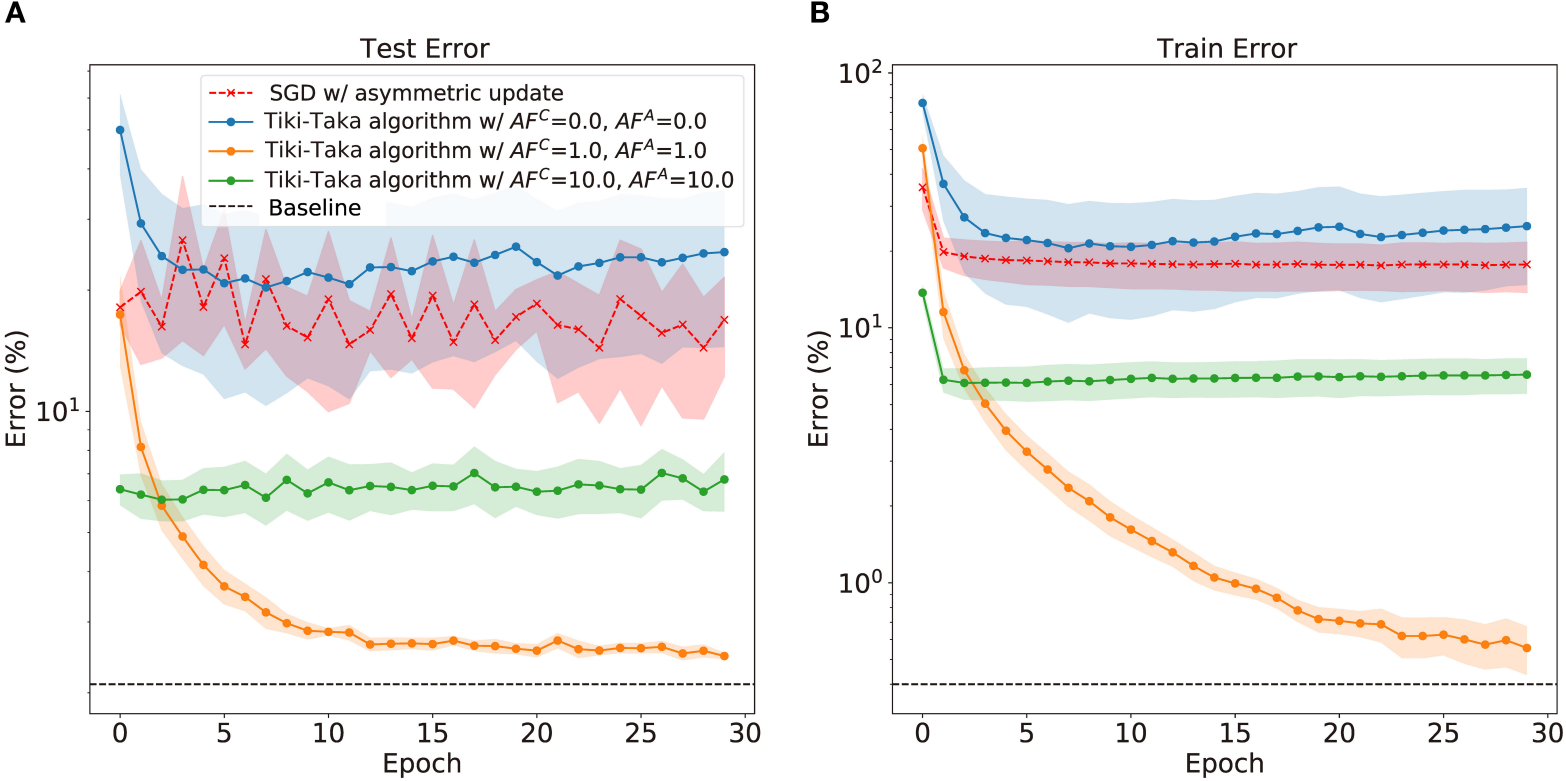
The train error **(A)** and test error **(B)** on handwritten digits images (MNIST) dataset with MLP. The baseline indicates the averaged error of the last 3 epochs with symmetric linear device, and the neural network is trained with SGD. In the case of SGD with asymmetric non-linear device, the error is averaged over various *AF* > 0. In the case of Tiki-Taka algorithm, the error is averaged over various pair of SGD and transfer learning rates. The shaded area indicates standard deviation.

Figure 2 displays the classification error in MNIST handwritten digit recognition problem as a function of training epoch for various scenarios of training algorithm and *AF* values. We perform the experimental simulation using the open-source toolkit *aihwkit* (Rasch et al., 2021) and further details about the experimental environment can be found in Supplementary Materials section 3.3. First, performing SGD with asymmetric devices results in severe accuracy drop both for the train and test datasets when compared with the software baseline. Since the update asymmetry leads to non-unity *MF* and consequent unfavorable behavior in weight update process, a sharp drop in performance is found during the training. On the other hand, the cases with Tiki-Taka algorithm and finite asymmetry show robust performance reaching to the floating point baseline (Gokmen and Haensch, 2020). As shown in Figure 2, when *AF*^*A*^ = *AF*^*C*^ = 1.0, the train and test error gradually decrease and can reach to the baseline. However, the remaining question is how much asymmetry Tiki-Taka algorithm can tolerate. Other cases shown in Figure 2 with various pairs of *AF*^*A*^ and *AF*^*C*^ answer the question. The train error does not gradually decrease, which indicates that the weight vectors of neural networks are stuck at bad local optima. Interestingly, the case with *AF*^*A*^ = *AF*^*C*^ = 0 shows higher train and test error than those of SGD with asymmetric non-linear devices. This result indicates that there exist optimal pairs of *AF*, and a reliable method to determine the optimal pair of *AF*^*A*^ and *AF*^*C*^ is necessary. To find the method, it is necessary to quantify and understand how *MF* affects the optimizer, Tiki-Taka algorithm, during the neural network training.

## 4. Optimization based on History of Gradients

### 4.1. General Formulation for Gradient History-Based SGD Variants

Recent variants of SGD introduce the concept of history-dependent update and utilize gradient information at each step, including momentum and adaptive gradient, to overcome the issues of vanilla SGD (Kandel et al., 2020). In such methods, the weight update is determined not only by the current gradient calculation, but also by the history of previous gradients and their importance at the current step. For instance, if the current weight update includes only the first-order gradient, then the history dependent update is composed of first-order gradient vectors and their respective importance for each step.

** Definition 4.1 (Gradient Scheduler)**. *History-dependent optimization algorithm is generalized by the* *governing rules* *including* *n*^*th*^ *order gradients:*


(13) $$\begin{array}{l} w_{ij,T+1}\leftarrow w_{ij,T}-\Delta w_{ij,T}\\ \;=w_{ij,T}-\eta E_{t}[\text{sgn}(\nabla_{ij,t}L),\nabla_{ij,t}L,\cdots ,{\left(\nabla_{ij,t}L\right)}^{n}]. \end{array}$$

**t** = **t**(*k*) (*k* = 1, ⋯ , *T*) is a *gradient scheduler* or *scheduler*, *which contains information on the importance of the* *k*^*th*^ *gradients*, $sgn\left(\nabla_{ij}L\right),\nabla_{ij}L,\;\cdots \;,{\left(\nabla_{ij}L\right)}^{n}$.

Therefore, the scheduler, **t**(*k*), defines how the gradients of previous steps for *k* < *T* affect the update of current step at *k* = *T*, since **t**(*k*) contains information on weighting of the gradients dependent on the step, *k*.

### 4.2. Gradient Scheduler in Tiki-Taka Algorithm

From the Definition 4.1, we can find the **t**(*k*) for Tiki-Taka algorithm as follows (see Supplementary Materials section 2.2 for the detailed derivation):


(14) $$\begin{array}{l} \text{t}\left(k\right)=1_{T\equiv_{ns}0}\cdot {\text{u}}_{\pi }\left(i\right), \end{array}$$

where **1**_*T*_≡__*ns*_0_ is an indicator function which returns 1 every ns steps, **t**(*k*) is controlled by three hyper-parameters (Gokmen and Haensch, 2020): γ, *ns*, and **u**_π_. These parameters cause the weight matrix *W* to be updated sparsely and asynchronously.

**Figure 3:**
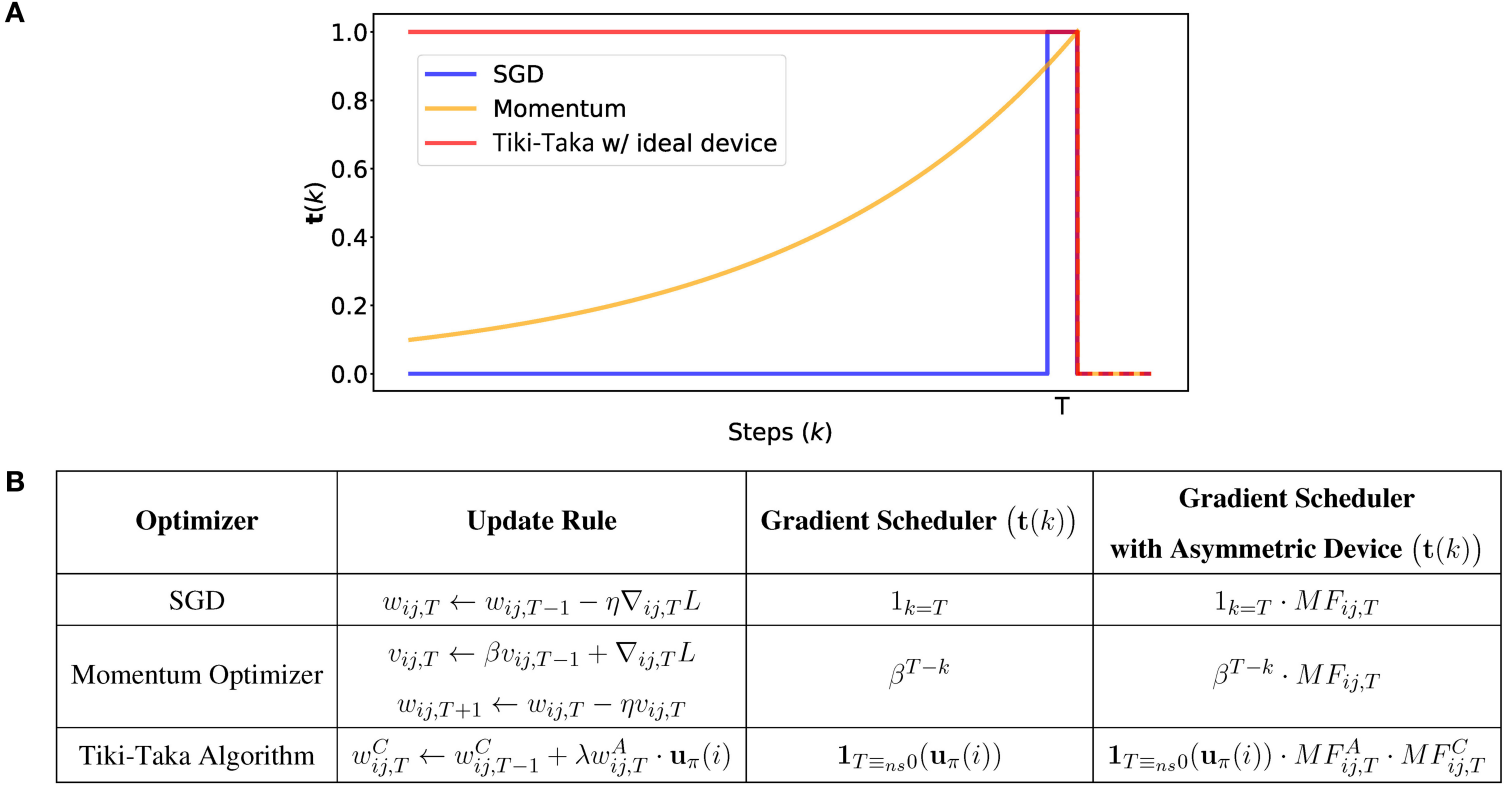
**(A)** Comparison of gradient scheduler, **t**(*k*), of SGD, momentum-based optimizer, and Tiki-Taka algorithm with respect to steps, k. **t**(*k*) is normalized by the value of **t**(*T*). **(B)** Summarized table of gradient scheduler of optimizers from **(A)**.

Figure 3 compares the gradient schedulers in three algorithms: vanilla SGD, momentum-based optimizer, and Tiki-Taka algorithm. The parameters of Tiki-Taka used are *ns* = 1, **u** = **1**, and γ = 0, and the ideal, symmetric and linear, switching characteristics are assumed for the devices in both arrays (i.e., $AF_{ij}^{A}=AF_{ij}^{C}=0$). With ideal devices, Tiki-Taka algorithm memorizes all the previous gradients with equal importance. Therefore, it resembles the momentum-based optimizer if β → 1. However, it should not be confused with the momentum-based optimizer, because the scheduler of Tiki-Taka algorithm with asymmetric devices depends not only on the step, but also on the history of gradients and slopes of devices unlike the momentum-based optimizer.

By including the update asymmetry, we can reformulate the scheduler of Tiki-Taka algorithm, Equation (14), as follows (see Supplementary Materials section 2.2) for the detailed derivation):


(15) $$\begin{array}{l} \text{t}\left(k\right)=1_{T\equiv_{ns}0}\left({\text{u}}_{\pi }\left(i\right)\right)\cdot MF_{ij,k}^{A}\cdot MF_{ij,T}^{C}. \end{array}$$

Equation (15) indicates that both *MF*^*A*^ and *MF*^*C*^ play an critical role in weight optimization. *MF*^*A*^ kicks in every step of *k*, and *MF*^*C*^ provides a factor until the last step of *T* in the scheduler of Tiki-Taka algorithm. Therefore, the optimizer of Tiki-Taka algorithm is uniquely determined by two *MF*'s. Considering that $w_{ij,T}^{A}$ is determined by the history of gradients, $\nabla L_{ij,1}^{\gamma },\cdots \;,\nabla L_{ij,T}^{\gamma }$, the scheduler of Tiki-Taka algorithm is parameterized by the step and the history of the first-order gradients. *MF*^*C*^ affects the optimization process for different *T, T*+1, ⋯  steps in common by scaling all the ideal **t**(*k*). Therefore, *MF*^*C*^ is one of key components in building the optimizer, and *MF*^*A*^ determines the optimizer of Tiki-Taka algorithm.

## 5. Linear Regression Analysis

**Figure 4:**
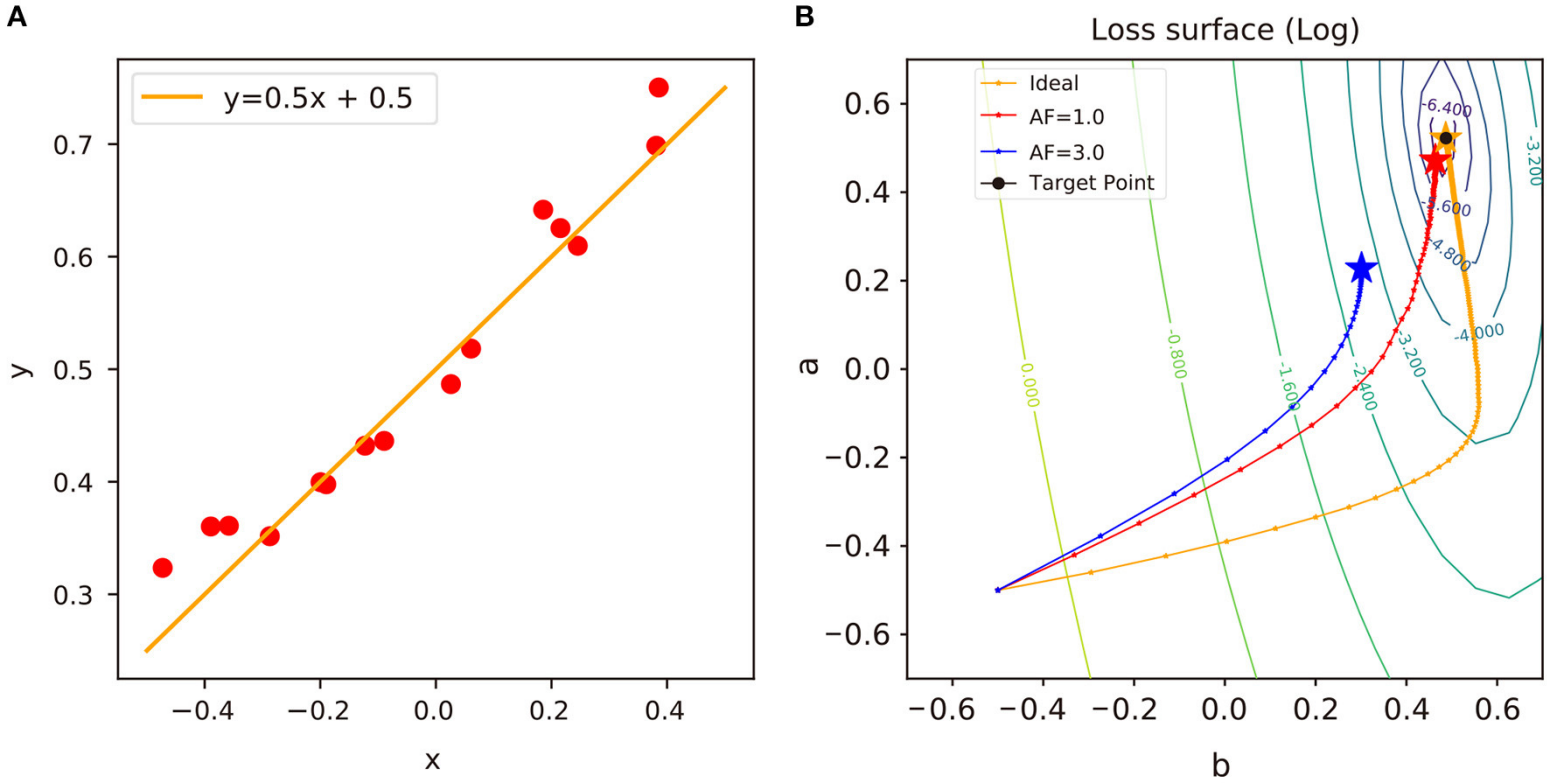
**(A)** An example of linear regression model and sampled data: *y* = *ax* + *b* + ϵ, where *a* ∈ *R* and ϵ~*N*(0, σ ^2^). In this example, *a* = 0.5, *b* = 0.5, *and* σ = 0.05. **(B)** Trajectories of weights in the log-scaled loss surface. A “target” point indicates a closed-form solution of linear regression model. i.e., (*a b*)^*T*^= (^*X*^*T*^*X*)−1^*X*^*T*^*y*, where *X* is the *N* × 2 input matrix and *y* is the *N* × 1 output vector. The “ideal” trajectory indicates a trajectory of a neural network optimized by vanilla SGD using ideal device. We denote it as “ideal” only in reference to the problem of convex optimization (see Supplementary Materials section 3.2 for the details of experimental environments).

To analyze the impact of update asymmetry in neural network training, we perform a series of linear regression experiments with different algorithms and display the results in Figure 4. With the presence of update asymmetry, the weight vectors of a neural network are stuck at sub-optima (Kim et al., 2019a) as shown in Figure 4B, and the linear regression is unable to approach the global optimum in this convex problem. Since the expected change of a weight around the sub-optima region over the given distribution of data *D*, **E**_*X*~*D*_[Δ*w*_*ij*_], is 0 on average, there is no room for the weight to progress further (Gokmen and Haensch, 2020). If the asymmetry factor of a crosspoint element, *AF*, is large, then the weight vector will converge to the region further away from the global optimum. This is because larger *AF* values cause larger variations in *MF* and equilibrium region to be formed away from the global optimum.

**Figure 5:**
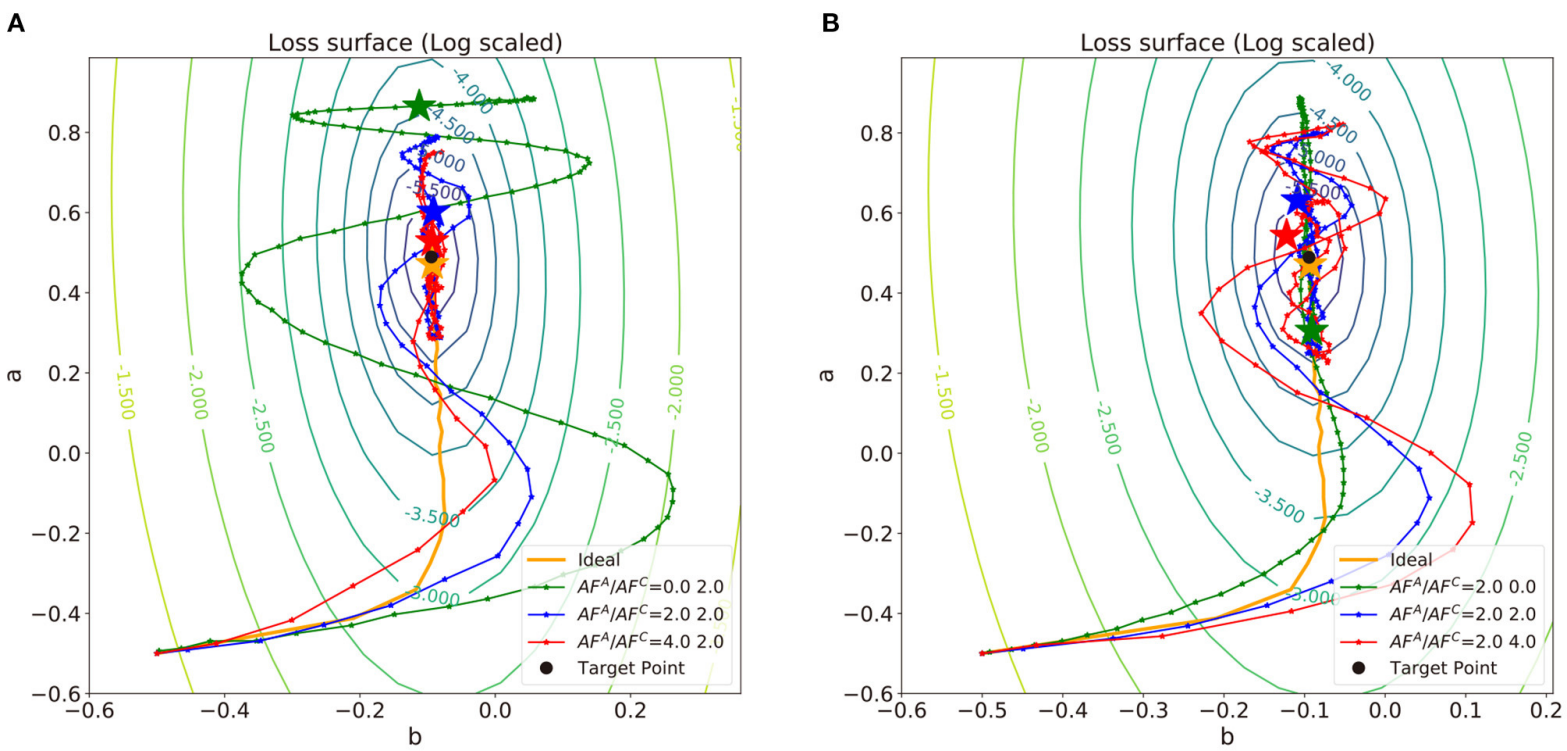
Trajectories of weights in the log-scaled loss surface for convex problem where the data is sampled from the same distribution as described in Figure 4A and the normalized schedulers, **t**(*k*), of a variable *b* described in Figure 4A. **(A)** *AF*^*C*^ is fixed with 2.0 and **(B)** *AF*^*A*^ is fixed with 2.0. The points of each trajectory are marked for every epoch.

In Figure 5, we repeat the linear regression experiments with Tiki-Taka algorithm and different combinations of *AF* values and observe the impact of update asymmetry on the linear regression results. Unlike the previous results with vanilla SGD, neural networks trained by Tiki-Taka algorithm with certain combinations of *AF*^*A*^ and *AF*^*C*^ are able to converge to the global optimum, even with the update asymmetry. Since Tiki-Taka algorithm can accumulate gradients of previous steps in the auxilary array and utilize the information for optimization, the weight vector can escape from the sub-optima dug by *MF* and converge to the global optimum.

**Figure 6:**
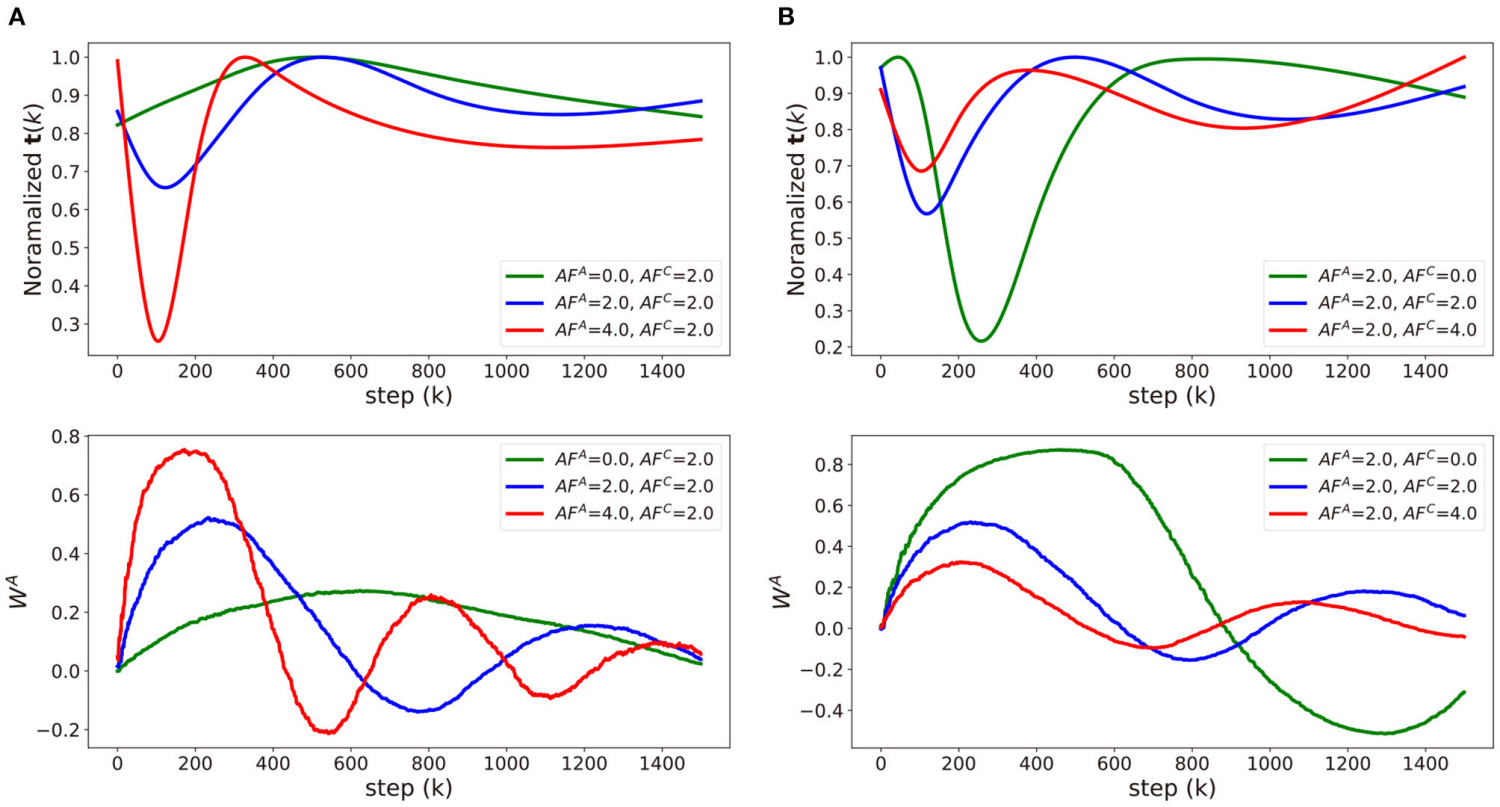
Comparisons of approximated **t**(*k*) and *W*^*A*^ of three cases in **(A)** Figure 5A and **(B)** Figure 5B. Raw data of **t**(*k*) and *W*^*A*^ was averaged using exponential moving average and approximated with natural smoothing spline method.

To analyze the impact of update asymmetry in the array A and C independently, we modulate *AF*^*A*^ (Figure 5A) and *AF*^*C*^ (Figure 5B) in an alternating fashion and compare the neural network training performance. The corresponding **t**(*K*) and *W*^*A*^ value as a function of training step for each case are visualized in Figure 6. Figure 5A shows the trajectories of weight vectors in three cases where *AF*^*A*^ values are 0.0, 2.0 and 4.0, respectively, and *AF*^*C*^ is fixed at 2.0. First, if *AF*^*A*^ = 0, then $MF_{ij,k}^{A}≃1$ at all steps *k*, which indicates that $\text{t}\left(k\right)≃MF_{ij,T}^{C}$. In this case, the large oscillation and deviation from the path by SGD are observed in the weight trajectory since the optimizer memorizes all the history of gradients with equal importance in the array *A* and the previous gradients $w_{ij}^{A}$ can only be changed slowly. On the other hand, when *AF*^*A*^ is 2.0 or 4.0, **t**(*k*) abruptly changes curvature near the 150^*th*^ step point as illustrated in Figure 6. If ∇_*ij*_*L* < 0 for *T*−1 steps, then $MF_{ij,k}^{A}\;\left(k<T\right)$ decreases as $w_{ij}^{A}$ increases as discussed in Definition 3.1. After the *T* steps, $MF_{ij,k}^{A}\;\left(k\geq T\right)$ abruptly increases as sgn(∇_*ij*_*L*) is inverted. The implication of the “abruptness" is that the optimizer is likely to forget the gradients of previous steps right before sgn(∇_*ij*_*L*) is inverted, and accept newly updated gradients. The “abruptness" amplifies with devices with larger *AF*^*A*^. In other words, the optimizer has an “easy come, easy go" memory that stores the history of gradients, and **t**(*k*) determines the speed of accumulating and forgetting the gradients. This behavior resembles that of the first order momentum optimizer. However, Tiki-Taka algorithm decides to decay the gradients by accumulating the successive gradients of same sgn(∇_*ij*_*L*) while the first order momentum optimizer merely decays the gradients far from the current step. In Figure 5, the trajectories with large *AF*^*A*^ are less inclined to oscillate and deviate as the optimizer forgets the information right before sgn(∇_*ij*_*L*) is inverted. It helps the weight vector to converge to the global optimum. However, it also fades out the regularization effect. Thus, there exists optimal range of *AF*^*A*^ that guarantees convergence and regularization.

Modulating *AF*^*C*^ impacts differently on the weight vector trajectories as shown in Figure 5B, where the trajectories of weight vectors with *AF*^*C*^ = 0.0, 2.0, and 4.0 are displayed while *AF*^*A*^ is fixed at 2.0. As *AF*^*C*^ increases, the weight update becomes less frequent with larger update steps, and, consequently, the frequency and magnitude of the oscillation in the weight vector becomes larger. This observation is expected from the fact that $\text{V}a\text{r}\left[\Delta w_{ij}^{C}\right]\propto {\left(AF_{ij}^{C}\right)}^{2}$ and $\text{E}\left[\Delta w_{ij}^{C}\right]\propto AF_{ij}^{C}$. With large *AF*^*C*^, the array *A* value fluctuates by frequently changing signs as shown in Figure 6 and cannot drive *W*^*C*^ in a consistent manner, which leads to a large oscillation in *W*^*C*^. In that sense, array *A* can capture the gradient history better when *AF*^*C*^ is smaller. However, with larger *AF*^*C*^, $MF_{k}^{C}$ gradually decreases as a function of step *k*, if the signs of $W_{k}^{A}$ are the same. This observation shows the similar effects of the adaptive gradient (Duchi, 2011; Zeiler, 2012; Kingma and Ba, 2014), which can help the weight vector to converge by decreasing the amount of updates around the global optimum when *AF*^*C*^ is set within the proper range. Therefore, the impact of *AF*^*A*^ and *AF*^*C*^ becomes highly entangled during optimization process, and it is a challenging task to find the optimal *AF* value for each array. A systematic method to find the optimal values of *AF*^*A*^ and *AF*^*C*^, which are highly dependent on the specific dataset and neural network architecture, is required for the convergence and performance of the neural network training.

## 6. Impact of Update Asymmetries on Neural Network Performance

### 6.1. Experimental Results

As we discussed in the previous sections, the update asymmetry in array *A* and *C* plays an important role in neural network training with Tiki-Taka algorithm as an optimizer. To experimentally explore the impact of *AF*^*A*^ and *AF*^*C*^ on training neural networks, we perform a series of experiments by varying *AF*^*A*^ and *AF*^*C*^ values and training a multi-layer perceptron (MLP) with MNIST dataset. For this experiment, we set (*w*_*min*_, *w*_*max*_) = (–1,1) and $\Delta {\hat{w}}_{0}=0.001$ for all devices in the array as mentioned in section 2.2, which illustrates the device reaching *w*_*max*_ = 1 from initial weight value of *w*_*min*_ = −1 within 600 updates if ($x_{i}^{q}$, $\delta_{j}^{q}$) = (1, –1) is given during update phase (see Supplementary Materials section 3.2 for the details of experimental environments).

**Figure 7:**
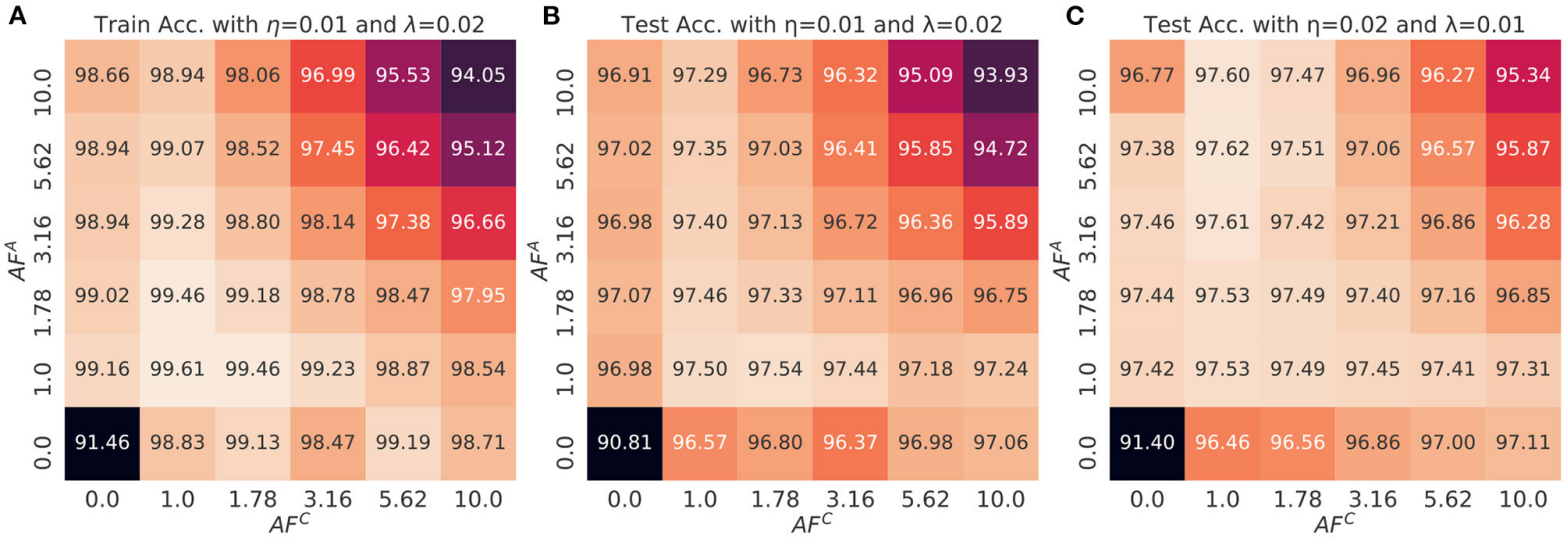
The **(A)** train and **(B,C)** test accuracy of a multi-layer perceptron as a function of *AF*^*A*^ and *AF*^*C*^. The displayed values are averaged over the last three epochs of the training and also three different initialization conditions. The mini-batch size is 1. The transfer learning rate and SGD learning rate is (0.01 and 0.02) for **(B)** and (0.02 and 0.01) for **(C)**.

Figure 7 shows the heat map of train (Figure 7A) and test accuracies of MLP as a function of *AF*^*A*^ and *AF*^*C*^ values and with two different set of SGD and transfer learning rates (Figures 7B,C). The results show the consistent observations as analyzed in section 5. First, when *AF*^*C*^ is fixed, the train and test accuracies peak at *AF*^*A*^ = 1.78 and decrease as it becomes larger or smaller. Smaller *AF*^*A*^ causes the optimizer to accumulate the history of gradient with equal importance, which hampers the convergence of the weight vectors due to oscillation. However, this effect is reduced with larger *AF*^*C*^ thanks to the adaptive gradient impact. In contrary, larger *AF*^*A*^ values disallow accumulation of the full history of gradients, which results in reduction of the regularization effect. This tendency is more significant for larger *AF*^*C*^ values, where the variance and expectation of updates for the array *C* is large enough not to store the entire history of gradients. Considering that larger *AF*^*A*^ causes memorization of short-term gradients, the regularization effect becomes weakened.

### 6.2. Impact of Learning Rates and Robustness Score

The optimal combination of *AF* values, which provide the best accuracy, can be changed when the combination of learning rates vary. For example, in Figure 7B, the test accuracy peaks at the combinations, (*AF*^*A*^, *AF*^*C*^) ∈ {(1.0, 1.0), (1.78, 1.0), (1.0, 1.78)} when the transfer learning rate and SGD learning rate is (0.01, 0.02). However, as shown in Figure 7C, the combination of *AF* is optimal when (*AF*^*A*^, *AF*^*C*^)∈{(3.16, 1.0), (5.62, 1.0), (10.0, 1.0)} if the neural network is trained with different pair of learning rates, (0.02, 0.01). Considering that *AF* of each device is typically pre-determined at the moment of device fabrication, it is crucial to find the robust *AF*^*A*^ and *AF*^*C*^ values over the learning rate space which do not degrade the accuracy. From this perspective, finding the robust region of *AF* without searching the best pair of learning rates can be guaranteed by introducing the thresholds of certain measurements such as test accuracy. The following *robustness score* provides the hard-bound threshold for finding the optimal combination of *AF* over the space of learning rates.

** Definition 6.1 (Robustness score)**. Suppose that *m* is a neural network model, and *Meas*(·) is a measurement such as accuracy or loss of the model *m* after training for a given dataset *D*. H is a hyper-parmeter space for software training, H={(η,λ), where η = SGD learning rate andλ = transfer learning rate}. A *Robustness score*, *RS*(*m*), for the given threshold, *th*, is defined as follows:


(16) $$\begin{array}{l} RS\left(m\right)=\underset{h\in H}{\sum }1\left(Meas\left(m\left(h\right)\right)>th\right) \end{array}$$

**Figure 8:**
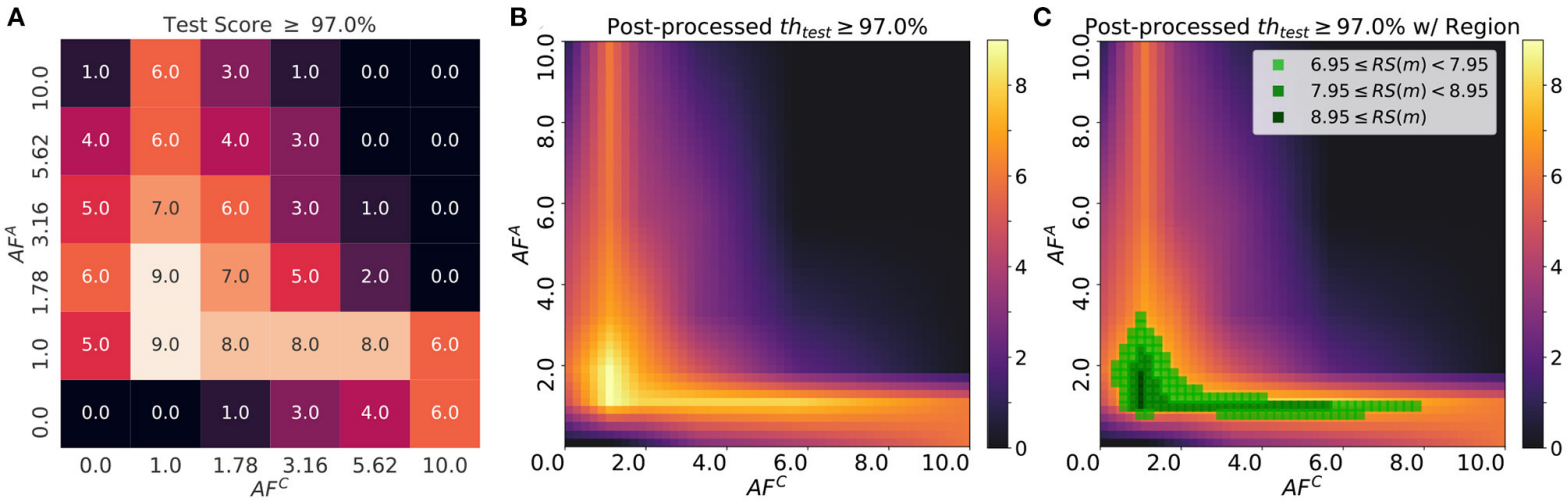
**(A)** The robustness score based on the test accuracy with a given threshold, 97.0%, where the number indicates how many cases with the test accuracy over the threshold. *dw*_*min*_ is set at 0.001. **(B)** The post-processed robustness score, where the step size of each domain in *AF* is 0.2 and σ = 0.2. **(C)** The regions with high robustness score are highlighted to visualize the area where the test accuracy is universally high as the learning rates change.

Figure 8 illustrates the robustness score, *RS*(*m*), over a learning rate space, H={(η,λ)}, where η∈{0.01, 0.02, 0.04} and λ∈{0.01, 0.02, 0.04}. As discussed in section 3.2, *AF* of each array affects the test accuracy of the network using Tiki-Taka algorithm. Test accuracies do not reach sufficient value when AF of each array is too small (*AF*^*A, C*^ = 0) or large (*AF*^*A, C*^ = 10). Therefore, optimal *AF* value pairs which maximize the robustness score exist near *AF*^*A, C*^ = 1 as shown in Figure 8. The score map has two implications. First of all, if the minimum requirement of test accuracy (threshold) is determined, the region whose robustness scores are over a certain criteria could be used to define the minimum specifications of update asymmetry. Therefore, one can obtain the range of *AF* value for each array which gurantees the minimum test accuracy. Second, by scanning over various pairs of learning rates, one can identify the region which provides the *AF* values which are most independent on the learing rates. To find such region, the score is post-processed with interpolation and gaussian filtering. The robustness score is approximated by piece-wise linear interpolation in the domain of *AF*^*A*^ and *AF*^*C*^. The interpolated data is smoothed by gaussian filtering (*g*(*AF*^*A*^, *AF*^*C*^))) to obtain the robust region in the domain: *g*(*AF*^*A*^, *AF*^*C*^) = (2π^σ^2^)−1/2^·*exp*(−(((*A*^*F*^*A*^)2^+(*A*^*F*^*C*^)2^)/(2σ^2^)), where we define the degree of “robustness" of the score map by modulating σ of a gaussian filter. Finding the robust region with a gaussian filter is consistent with the definition of variations in *AF* of devices. In Figure 8C, the robust region is around *AF*^*A*^ = 1.2 and *AF*^*C*^ = 1.0 with the score greater than 8.95. As we experiment with a total of 9 scenarios of learning rates, the region with the score greater than 8.95 displays the required test accuracy within the given range of learning rate pairs. When compared to the region with *RS*(*m*) = 4.5, the region with *RS*(*m*)≥8.95 shows almost doubled efficiency during on-device training. In other words, it requires half of the resources to find the optimal pair of learning rates. This calibration method to find the best combination of *AF* is applicable to other types of hyper-parameters if the hyper-parameters space H can be expanded to include them.

### 6.3. Inspection on Optimal Range of Slope for Different Thresholds

**Figure 9:**
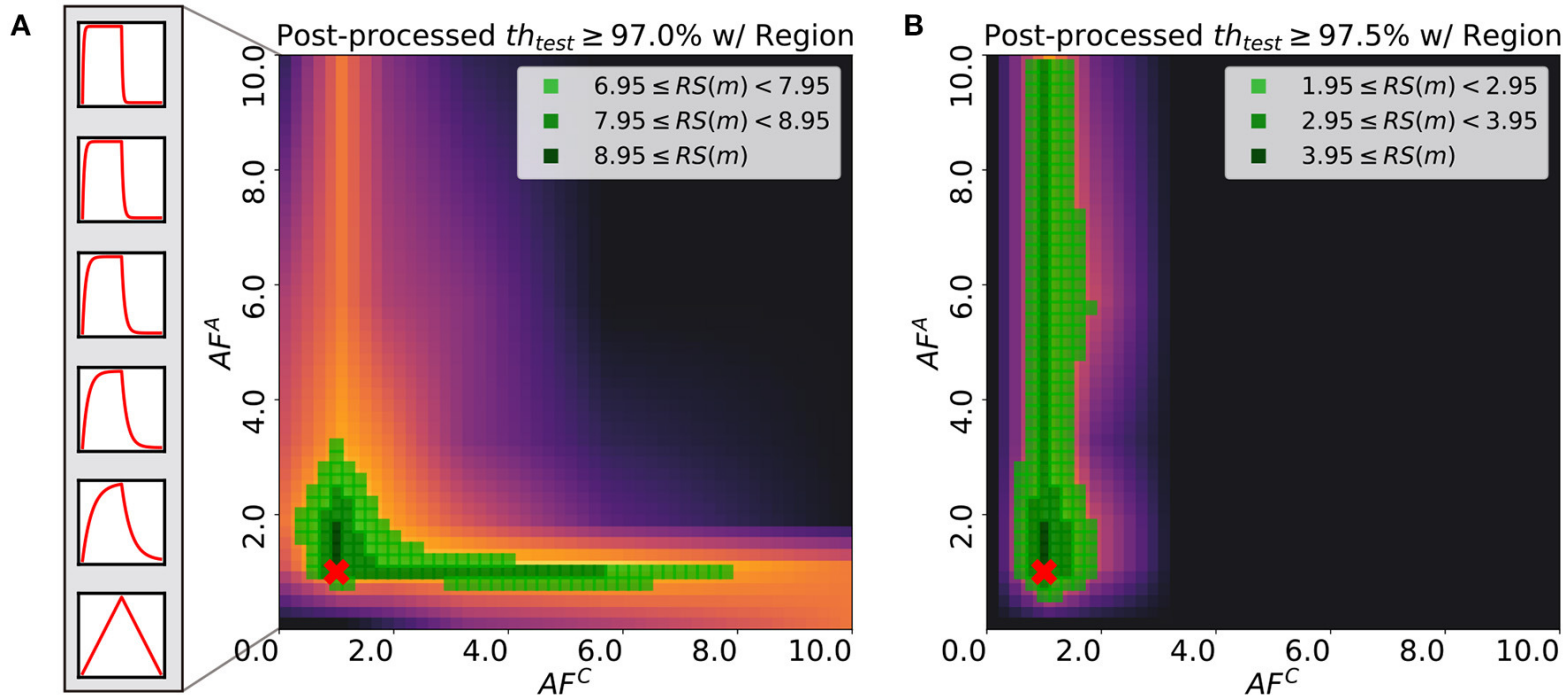
Post-processed test robustness score on various thresholds: **(A)** 97.0% and **(B)** 97.5%. The green-colored regions are highlighted with different light and shade. Red cross point indicates the pair of asymmetric factors for baseline model used in original Tiki-Taka algorithm (Gokmen and Haensch,2020). Boxes on the left side of **(A)** show the asymmetry of device response in accordance with each asymmetric factor.

In practical applications, the target performance of the network, such as the test accuracy of the MNIST classification task using MLP, is one of the most significant design choices as some tasks require the maximum accuracy while others do not. Considering that the robustness score, *RS*(*m*), varies as target accuracy changes, studying the relationship between *RS*(*m*) and the target threshold value can provide insights on the choice of *AF* for each array. Figure 9 illustrates the robustness score maps with two different threshold values, 97.0 and 97.5%. When the threshold is set to 97.0%, the score is robust against the change in *AF*^*C*^ value and the efficiency of searching over the hyper-parameter space, H with *AF*^*A*^ is not degraded in the proper region. However, in the case of the threshold being 97.5%, the best choice is to fix *AF*^*C*^ around 1.0. Then the asymmetry requirement for the array A is lifted. Although the robustness scores of the case with the threshold being 97.5% are relatively lower than those of 97.0%, selecting *AF*^*A*^ and *AF*^*C*^ of the region has comparative advantages over the other regions.

Consistent with the analysis in sections 5 and 6.1, Figure 9A describes that the neural networks are able to find the optimum with a wide range of *AF*^*C*^ values over H if *AF*^*A*^ is in the proper region. This observation is due to the adaptive gradient effect that *AF*^*C*^ brings, which helps the convergence around the nearest local optimum. In this case, the neural network converges to the local optimum instead of further exploring the loss surface. This results in the high robustness score region along the wide range of *AF*^*C*^, though it does not guarantee higher accuracy. In Figure 9B, on the other hand, the region satisfying over 97.5% accuracy is spread along the axis of *AF*^*A*^, which indicates that the specification for *AF*^*C*^ value is strict. If *AF*^*C*^ value is within the specification, then the robustness score depends on the choice of *AF*^*A*^. With smaller *AF*^*A*^ values, the optimizer allows the weights of neural networks to explore over loss surface, which enables to find better optimum.

### 6.4. Application to SNN

Recent research on SNN and its implementations using resistive memory devices have shown that the devices are designed explicitly to be embedded with the learning algorithm for SNN such as Spike-timing-dependent plasticity (STDP) and equilibrium propagation (Scellier and Bengio, 2017). Accordingly, the update asymmetry of devices is more likely to affect the process of training neural networks (Kwon et al., 2020). Inspired by this motivation, they demonstrated that asymmetric non-linear devices are more powerful than symmetric linear devices (Brivio et al., 2018, 2021; Kim et al., 2021). Our analytical tools and calibration method can be applied to illustrate the relationship between the update asymmetry of devices and its impact on training in detail. Therefore, it is worthwhile to pursue further studies on the relationship in more extensive range of cases of learning algorithms.

## 7. Conclusion and Discussion

In this work, the impact of update asymmetry in array *A* and *C* and learning rates on network performance when training neural networks with Tiki-Taka algorithm is quantitatively analyzed. We introduced the concept of *gradient scheduler*, which defines the weights of previous gradient values, to explain how the update asymmetry impacts on solving a convex problem. With update asymmetry, we derived that asymmetry factor involved in *gradient scheduler* that the training of neural networks is affected. As we inspected on the gradient schedulers, larger asymmetry factor of *A* accelerates accumulating and forgetting the history of gradients while that of *C* brings adaptive gradient effects. We showed that the update asymmetry levels in the main and auxiliary arrays impact differently on training neural networks, indicating that requirements for asymmetry on each array are different. We also proposed a novel calibration metric, *Robustness Score*, to quantify and visualize the performance of the network as a function of device asymmetry and network parameters with respect to the target accuracy threshold. By searching over the hyper-parameter space of Tiki-Taka algorithm, we quantified the specification of device asymmetry for *A* and *C* arrays depending on the target accuracy value of choice. Our analysis shed light on further relaxing device specifications for the realization of analog neural network accelerators in hardware and provide a guideline to realize the resistive switching devices with robust training performance over the space of hyper parameters.

## Data Availability Statement

The raw data supporting the conclusions of this article will be made available by the authors, without undue reservation.

## Author Contributions

SK conceived the original idea. CL formulated the theory. CL, KN, and WJ developed methodology and conducted experiments. CL, TG, and SK analyzed and interpreted results. All authors drafted and revised the manuscript.

## Funding

This work was supported by Samsung Science & Technology Foundation (grant no. SRFC-IT2001-06).

## Conflict of Interest

CL is employed by NAVER Clova, South Korea. TG is employed by IBM Research, USA. The remaining authors declare that the research was conducted in the absence of any commercial or financial relationships that could be construed as a potential conflict of interest.

## Publisher's Note

All claims expressed in this article are solely those of the authors and do not necessarily represent those of their affiliated organizations, or those of the publisher, the editors and the reviewers. Any product that may be evaluated in this article, or claim that may be made by its manufacturer, is not guaranteed or endorsed by the publisher.
