# Supplementary material for: Impact of Asymmetric Weight Update on Neural Network Training With Tiki-Taka Algorithm
Source: Front Neurosci. 2022 Jan 6;15:767953. doi: 10.3389/fnins.2021.767953 (PMC8770851; doi:10.3389/fnins.2021.767953)
Supplement: Supplementary file 1 [file Data_Sheet_1.PDF]

Supplementary Material

1 DEVICE MODELING AND SGD BY FUNCTION DECOMPOSITION

1.1 Device Assumption

In *aihwkit* (Rasch et al., 2021), we use *LinearStepDevice* to model a device whose deviation of conductance response ($\triangle g$) is assumed to be linear. With this model, the deviation of conductance differs for two cases: potentiation (denoted as p) and depression (denoted as d).

$$\begin{aligned} \triangle g_{ij}^{p} &= AF_{ij}(-\widehat{s}_{ij} g_{ij} + \triangle \widehat{g}_{0,ij}) \\ \triangle g_{ij}^{d} &= AF_{ij}(\widehat{s}_{ij} g_{ij} + \triangle \widehat{g}_{0,ij}) \end{aligned} \tag{S1}$$

During the potentiation and depression, the device is naturally bounded if $\triangle g_{ij}^{p} = 0$ and $\triangle g_{ij}^{d} = 0$, respectively. Therefore, the upper and lower bound of the conductance, g_{ij}, are $g_{max,ij} = \dfrac{\triangle \widehat{g}_{0,ij}}{\widehat{s}_{ij}}$ and $g_{min,ij} = -\dfrac{\triangle \widehat{g}_{0,ij}}{\widehat{s}_{ij}}$ $(s_{ij} \neq 0)$.

$$g_{min,ij} = -\frac{\triangle \widehat{g}_{0,ij}}{\widehat{s}_{ij}} \leq g_{ij} \leq \frac{\triangle \widehat{g}_{0,ij}}{\widehat{s}_{ij}} = g_{max,ij} \tag{S2}$$

With this assumption, all the devices having different AF_{ij} are bounded under allowed range. If $\widehat{s}_{ij} = 0$, the device is not naturally bounded. Therefore, the conductance is clipped to satisfy the allowed range. By multiplying K with the conductance, the weight, w_{ij}, is bounded in the range of $\Big[-\dfrac{\triangle \widehat{w}_{0,ij}}{\widehat{s}_{ij}}, \dfrac{\triangle \widehat{w}_{0,ij}}{\widehat{s}_{ij}} \Big]$.

1.2 Function Decomposition

To integrate the weight (Equation 4) and conductance update rule (Equation 5 and 6), $\triangle w_{ij,k}$ which includes non-linear asymmetry of a device can be approximately reformulated as follows:

$$\begin{aligned} \triangle w_{ij,k} = K \triangle g_{ij,k} &= K \cdot d_{ij} \cdot (s_{ij} \cdot g_{ij,k} + \triangle g_{0,ij}) \\ &= K \cdot \text{round}\Big(\frac{\eta \nabla_{ij,k} L}{K \triangle \widehat{g}_{0,ij}}\Big) \cdot (s_{ij} \cdot g_{ij,k} + \triangle g_{0,ij}) \\ &\simeq \eta \nabla_{ij,k} L \frac{K(s_{ij} \cdot g_{ij,k} + \triangle g_{0,ij})}{K \triangle \widehat{g}_{0,ij}} = \eta \nabla_{ij,k} L \frac{\triangle g_{ij,k}}{\triangle \widehat{g}_{0,ij}} \end{aligned} \tag{S3}$$

There are two cases for weight update rule in accordance with the sign of $\nabla_{ij,k} L$ where if the sign is positive, then it corresponds to potentiation and otherwise, depression.

$$\begin{cases} w_{ij,k+1} \leftarrow w_{ij,k} - \eta \nabla_{ij,k} L \dfrac{\triangle g_{ij,k}^{p}}{\triangle \widehat{g}_{0,ij}}, \ \text{if } \nabla_{ij} L > 0 & \text{(S4)} \\ w_{ij,k+1} \leftarrow w_{ij,k} - \eta \nabla_{ij,k} L \dfrac{\triangle g_{ij,k}^{d}}{\triangle \widehat{g}_{0,ij}}, \ \text{otherwise.} & \text{(S5)} \end{cases}$$

By even-odd function decomposition, the above equation can be represented as follows (Gokmen and Haensch, 2020):

$$w_{ij,k+1} \leftarrow w_{ij,k} - \eta \nabla_{ij,k} L \cdot \frac{1}{2} \cdot \frac{\triangle g^p_{ij,k} + \triangle g^d_{ij,k}}{\triangle \widehat{g}_{0,ij}} - \eta |\nabla_{ij,k} L| \cdot \frac{1}{2} \cdot \frac{\triangle g^p_{ij,k} - \triangle g^d_{ij,k}}{\triangle \widehat{g}_{0,ij}} \tag{S6}$$

From the above equation, denote the even part as $Sym(w_{ij,k})$ and the odd part as $Asym(w_{ij,k})$. With the device model, these two functions are simplified by the function of AF_{ij} and $w_{ij,k}$:

$$\begin{aligned}
Sym(w_{ij,k}) &= \frac{1}{2} \cdot \frac{\triangle g^p_{ij,k} + \triangle g^d_{ij,k}}{\triangle \widehat{g}_{0,ij}} \\
&= \frac{AF_{ij} \cdot \widehat{g}_{0,ij}}{2\triangle \widehat{g}_{0,ij}} \Big((\frac{1}{g_{max,ij}} \cdot g_{ij} + 1) + (-\frac{1}{g_{max,ij}} \cdot g_{ij} + 1) \Big) = AF_{ij} \\
Asym(w_{ij,k}) &= \frac{1}{2} \cdot \frac{\triangle g^p_{ij,k} + \triangle g^d_{ij,k}}{\triangle \widehat{g}_{0,ij}} \\
&= \frac{AF_{ij} \cdot \widehat{g}_{0,ij}}{2\triangle \widehat{g}_{0,ij}} \Big((\frac{1}{g_{max,ij}} \cdot g_{ij} + 1) - (-\frac{1}{g_{max,ij,k}} \cdot g_{ij} + 1) \Big) = AF_{ij} \frac{g_{ij}}{g_{max,ij}} \\
&= AF_{ij} \frac{w_{ij}}{w_{max,ij}}
\end{aligned} \tag{S7}$$

From Definition 3.1, if $Sym(w_{ij,k}) = 0$, we call the device as *symmetric linear device*. Otherwise, we it is called as the *asymmetric non-linear device*. As for the *symmetric linear device*, the amount of update is consistent that the conductance gradually increases or decreases.

2 GRADIENT SCHEDULER

2.1 Derivation of gradient scheduler of different optimization algorithm and Mismatch Factor

Following the Definition 4.1, the scheduler of vanilla SGD, $\mathbf{t}(k)$ $(k = 1, ..., T)$, can be expressed as a simple indicator:

$$\mathbf{t}(k) = \mathbf{1}_{k=T} \tag{S8}$$

If $k \neq T$, then $\mathbf{t}(k) = 0$ and $\mathbf{t}(k) = 1$ only at $k = T$. This indicates that only the gradient of current step T participates in the weight update, and the previous gradients are not used in vanilla SGD.

To include the impact of the device model, we can introduce MF in Equation S8 as follows:

$$\mathbf{t}(k) = \mathbf{1}_{k=T} \cdot MF_{ij}(w_{ij,T}, \text{sgn}(\nabla L_{ij,T})) = \mathbf{1}_{k=T} \cdot MF_{ij,T} \tag{S9}$$

In the case of the first-order momentum-based optimization algorithm, the first momentum is dependent on the history of previous first-order gradients.

$$v_{ij,T} \leftarrow \beta v_{ij,T-1} + \nabla_{ij,T} L \tag{S10}$$

$$w_{ij,T+1} \leftarrow w_{ij,T} - \eta v_{ij,T} \tag{S11}$$

, where $v_{ij,0} = 0$ and β is a hyper-parameter to the ratio of importance between the weight change and the gradient. η is the learning rate of weight change. By developing Equation S11 for the entire steps, we draw

an equation that fits the governing rules:

$$
\begin{aligned}
w_{ij,T} - \eta v_{ij,T} &= w_{ij,T} - \eta(\beta v_{ij,T} + \nabla_{ij,T} L) \\
&= ... = w_{ij,T} - \eta \sum_{m=1}^{T} (\beta^{T-m} \nabla_{ij,m} L).
\end{aligned} \tag{S12}
$$

We can rewrite $\triangle w_{ij,T}$ and derive the scheduler, $\mathbf{t}(k)$, as follows:

$$
\triangle w_{ij,T} = \mathbf{E_t}[\nabla_{ij,\mathbf{t}} L] = \sum_{m=1}^{T} (\beta^{T-m} \nabla_{ij,m} L) \tag{S13}
$$

$$
\mathbf{t}(k) = \beta^{T-k} \tag{S14}
$$

, where $0 << \beta < 1$ is a decay factor to weigh on the importance between the immediate gradient and accumulated gradients. If we include the device model, it can be reformulated to be similar to Equation S9:

$$
\mathbf{t}(k) = \beta^{T-k} \cdot MF_{ij,T} \tag{S15}
$$

2.2 The Scheduler of Tiki-taka Algorithm

Using an indicator function, Equation 8 can be represented as follows:

$$
\begin{aligned}
w^C_{ij,k+1} &\leftarrow w^C_{ij,k} + \mathbf{1}_{k+1 \equiv_{ns} 0}(\lambda w^A_{ij,k+1} \cdot ((P_\pi^{\lfloor k+1/ns \rfloor} \mathbf{u})(i))) \\
&= w^C_{ij,k} + \lambda w^A_{ij,k+1} \cdot \mathbf{1}_{k+1 \equiv_{ns} 0}(\mathbf{u}_\pi(i))
\end{aligned} \tag{S16}
$$

, where P_π is a permutation matrix of π: $\{1, ..., n\} \rightarrow \{1, .., n\}$. i.e., $\mathbf{u}_\pi = P_\pi^{\lfloor k+1/ns \rfloor} \mathbf{u}$. For each step with the interval ns, w^C_{ij} is updated as follows. To simply notation, we denote $\mathbf{1}_{k+1 \equiv_{ns} 0}(\mathbf{u}_\pi(i))$ as$\mathbf{1}_{k+1}(\mathbf{u}_\pi(i))$:

$$
\begin{aligned}
& w^C_{ij,k} + \lambda w^A_{ij,k+1} \cdot \mathbf{1}_{k+1}(\mathbf{u}_\pi(i)) \\
&= w^C_{ij,k} + \lambda(w^A_{ij,k} - \eta \nabla^\gamma_{ij,k+1} L) \cdot \mathbf{1}_{k+1}(\mathbf{u}_\pi(i)) \\
&= w^C_{ij,k} + \lambda((w^A_{ij,k-1} - \eta \nabla^\gamma_{ij,k-1} L - \eta \nabla^\gamma_{ij,k} L) \cdot \mathbf{1}_{k+1}(\mathbf{u}_\pi(i)) \\
&= ... = w^C_{ij,k} - \lambda\eta\Big(\sum_{m=1}^{k} \nabla^\gamma_{ij,m} L\Big) \cdot \mathbf{1}_{k+1}(\mathbf{u}_\pi(i))
\end{aligned} \tag{S17}
$$

With non-ideal devices, by introducing MF, Equation S17 is reformulated as follows:

$$
\begin{aligned}
& w^C_{ij,k+1} \leftarrow w^C_{ij,k} + \lambda w^A_{ij,k+1} \cdot \mathbf{1}_{k+1} \\
& \quad = w^C_{ij,k} - \lambda\eta\Big(\sum_{m=1}^{k} \nabla^\gamma_{ij,m} L \cdot MF^A_{ij,m}(w^A_{ij,m}, \nabla L^\gamma_{ij,m})\Big) \cdot \mathbf{1}_{k+1} \cdot MF^C_{ij,k+1}(w^C_{ij,k+1}, \nabla L^\gamma_{ij,k+1})
\end{aligned} \tag{S18}
$$

With same method used in Supplement Sec. 2.1, we can rewrite $\triangle w_{ij,T}$ of tiki-taka algorithm and derive the scheduler, $\mathbf{t}(k)$, as follows:

$$\begin{aligned}\triangle w_{ij,T} &= \mathbf{E_t}[\nabla_{ij,\mathbf{t}}L] \\ &= \sum_{m=1}^{k} \nabla^{\gamma}_{ij,m}L \cdot \mathbf{1}_{k+1} \cdot MF^{A}_{ij,m}(w^{A}_{ij,m}, \nabla L^{\gamma}_{ij,m}) \cdot MF^{C}_{ij,k+1}(w^{C}_{ij,k+1}, \nabla L^{\gamma}_{ij,k+1})\end{aligned} \tag{S19}$$

$$\mathbf{t}(k) = \mathbf{1}_{k+1} \cdot MF^{A}_{ij,m}(w^{A}_{ij,m}, \nabla L^{\gamma}_{ij,m}) \cdot MF^{C}_{ij,k+1}(w^{C}_{ij,k+1}, \nabla L^{\gamma}_{ij,k+1}) \tag{S20}$$

3 EXPERIMENTAL ENVIRONMENT

We conduct several experiments using *aihwkit* (Rasch et al., 2021) and PyTorch (Paszke et al., 2019). Our simulation environment is as follows: Intel Xeon 4210R and 4214R; NVIDIA RTX 6000 and 3090.

3.1 Device Modeling Examples

To show the conductance response, we assume devices to be modeled as follows: $\triangle w_{0,ij} = 5 \cdot 10^{-5}$, $s_{ij} = 5 \cdot 10^{-5}$. The devices are updated with unit pulse generated by SPG during potentiation and depression.

3.2 Linear Regression

For linear regression, we build a linear model which has two parameters, a and b. Each parameter is implemented with *LinearStepDevice*. For implementing Tiki-taka algorithm, we use *TransferCompound* which is composed of a pair of *LinearStepDevice*. We assume the device to be modeled as follows: $\triangle w_{0,ij} = 1.66 \cdot 10^{-3}$; $s_{ij} = 1.66 \cdot 10^{-3}$. For the specifications of peripheral circuits and systems, we assume them as follows: DAC/ADC resolution=256; Bit length of SPG=100; Pulse type of SPG=Stochastic pulse. For the experiment in Figure 4, $N = 10$ and the mini-batch size is 1. The linear model is optimized by SGD for 200 epochs with learning rate as 0.04. For the experiments of Figure 5, $N = 15$ and the mini-batch size is 1. In this case, the linear model is optimized by Tiki-taka algorithm with SGD learning rate as 0.02 and transfer learning rate as 0.01.

3.3 MNIST Classification

For MNIST classification, the neural network has 3 hidden layers (784-512-256-128-10) and sigmoid function as an activation function. We use the cross entropy loss as a loss function and apply softmax at the output layer to normalize the output. The input data is normalized in $[0.0, 1.0]$. We assume the devices to be modeled as follows: $\triangle\widehat{w}_{0,ij} = 1.0 \cdot 10^{-3}$; $\widehat{s}_{ij} = 1.0 \cdot 10^{-3}$. For the specifications of peripheral circuits and systems, we assume them as follows: DAC/ADC resolution=256; Bit Length of SPG = 100; Pulsed Type=Stochastic pulse. We collect experimental results while modulating SGD (η) and transfer learning rate (λ), and also AF^A and AF^C as described in Sec. 6.2. In total, there are 324 cases. The neural network is trained over 30 epochs and the mini-batch size is 1. The results are averaged over 3 differently initialized MLP. Figure 2, 7, and 8 uses the experimental results.

REFERENCES

Gokmen, T. and Haensch, W. (2020). Algorithm for training neural networks on resistive device arrays. *Frontiers in neuroscience* 14

Paszke, A., Gross, S., Massa, F., Lerer, A., Bradbury, J., Chanan, G., et al. (2019). Pytorch: An imperative style, high-performance deep learning library. In *Advances in Neural Information Processing Systems 32*, eds. H. Wallach, H. Larochelle, A. Beygelzimer, F. d'Alché-Buc, E. Fox, and R. Garnett (Curran

Associates, Inc.). 8024–8035

Rasch, M. J., Moreda, D., Gokmen, T., Gallo, M. L., Carta, F., Goldberg, C., et al. (2021). A flexible and fast pytorch toolkit for simulating training and inference on analog crossbar arrays. *arXiv preprint arXiv:2104.02184*
